# HPV vaccination among ethnic minorities in the UK: knowledge, acceptability and attitudes

**DOI:** 10.1038/bjc.2011.272

**Published:** 2011-08-09

**Authors:** L A V Marlow

**Affiliations:** 1 Department of Epidemiology and Public Health, Cancer Research UK Health Behaviour Research Centre, UCL, Gower Street, London WC1E 6BT, UK

**Keywords:** review, ethnic, vaccination, cervical cancer, knowledge

## Abstract

**Background:**

Human papillomavirus (HPV) vaccination offers a unique opportunity for the primary prevention of cervical cancer. Studies suggest that knowledge and attitudes about the vaccine are likely to influence uptake. One limitation of most studies assessing HPV vaccine knowledge, attitudes and acceptability is their under representation of ethnic minorities. It is important to ensure that our understanding of HPV knowledge and attitudes include all ethnic groups in the UK. This article reviews research that has considered knowledge, acceptability and attitudes about HPV and the HPV vaccine among ethnic minorities in the UK.

**Methods:**

Articles in Medline, CINAHL and PsycINFO (January 2000–March 2010) were searched.

**Results:**

A total of 17 UK-based papers examined knowledge, attitudes or acceptability related to HPV vaccination in the ‘lay’ population (parents, adolescents or the general population as opposed to health professionals) and reported findings by ethnicity.

**Conclusion:**

Findings seem to suggest lower awareness of HPV and lower acceptability of the vaccination, which could be important if they are reflected in uptake. More research is needed with ethnic minority groups, particularly in the context of the vaccination programme.

## Introduction

Each year the UK sees around 3000 new cases of cervical cancer and just under 1000 deaths (CRUK, 2007, 2008). Incidence of cervical cancer has been dramatically reduced by the cervical screening programme, which is estimated to save around 3000 lives a year (Peto *et al*, 2004). However, there are inequalities in cervical cancer, with higher rates in women from deprived backgrounds (Quinn *et al*, 2001; Coupland *et al*, 2007). Until recently no research had considered ethnic inequalities, but a report published by the National Cancer Intelligence Network in 2009 suggested that Asian and Black women over 65 years are at increased risk of cervical cancer, whereas Asian women under 65 have a lower incidence of the disease. Small numbers meant it was not possible to explore ethnic variation in mortality rates (NCIN, 2009). Differences in lifestyle, which lead to increased risk of human papillomavirus (HPV) exposure or persistence (e.g. number of sexual partners, smoking), or screening participation are thought to be the most likely reasons for inequalities in cervical cancer (Akers *et al*, 2007). In particular, research has suggested lower uptake of cervical screening among some ethnic groups (Webb *et al*, 2004) and a recent study found that this was the case even when controlling for a range of socioeconomic status (SES) variables (Moser *et al*, 2009).

In 2008, a national HPV vaccination programme was launched in the UK. The HPV vaccination offers protection against HPV types 16 and 18, which are responsible for around 70% of cervical cancer (Munoz *et al*, 2004). The vaccine is offered to all girls in school year 8 (12–13 years), as well as to all girls up to 18 years old as part of a catch-up campaign (until July 2011). In the majority of cases it is offered in schools but is sometimes offered through primary care. The HPV vaccination programme offers the potential to overcome inequalities in cervical cancer, but this relies on good uptake of the vaccination across all SES and ethnic groups. Overall uptake of all three doses of the vaccine for the first year of the immunisation programme in England (2008/2009) was 80% for 12–13-year olds and 32% for 17–18-year olds (Department of Health, 2010a), but coverage has not been reported separately for different ethnic or SES groups.

Despite the high uptake levels that are being reported, it is still important to consider the reasons why some adolescents do not get the HPV vaccination and the potential for inequalities in HPV vaccine uptake. There are many practical barriers that may stop adolescents from having HPV vaccination (e.g. absence from school, difficulties getting to the doctor's surgery), but beliefs and attitudes about vaccines are also likely to have an influence on the decision to accept a vaccine (Sturm *et al*, 2005). In addition good knowledge about HPV is important in order to ensure decisions about the vaccine are informed (Marteau *et al*, 2001). In the 5 years preceding its launch, several studies explored knowledge, attitudes and acceptability of HPV vaccination in the UK. Because most studies were carried out before the implementation of the vaccination programme their focus was on acceptability (intention to accept the vaccine) rather than uptake, but the findings have helped to establish an understanding of the socio-demographic and psychosocial factors, which may have a role in the decision to accept HPV vaccination. One limitation of these studies is their underrepresentation of ethnic minorities (i.e., those from non-white backgrounds). In 2001, 4.6 million people in the UK considered themselves to be from an ethnic group other than ‘white’ (ONS, 2004). This non-white population is made up of predominantly South Asian (from this point on South Asian will be referred to as Asian) (mostly Indian, Pakistani and Bangladeshi), Black (mostly Caribbean and African) and Chinese people, and people who consider themselves to be from mixed backgrounds. Across the UK, these groups account for just 8% of the population, but certain areas have much greater proportions than others (Dobbs *et al*, 2006).

The literature on uptake of childhood vaccinations suggests that uptake varies by ethnicity, with the highest rates among Asian parents, followed by White and Black parents (Baker *et al*, 1984; Hawker *et al*, 2007). When making the distinction between children who are partially vaccinated and those who are unvaccinated, it appears that partial vaccination is associated with living in disadvantaged and ethnically dense areas, whereas parents of unvaccinated children are more highly qualified and more likely to be from Black Caribbean backgrounds (Samad *et al*, 2006). In addition, it appears that the drop in MMR uptake, following its suggested link with autism, was less prominent in non-white parents (Hawker *et al*, 2007). These findings suggest that some minority groups have lower and some have higher uptake of childhood vaccinations. However, we cannot assume that HPV vaccination will be seen in the same way as other childhood vaccinations. The Cancer Reform Strategy highlights the importance of reducing ethnic inequalities in cancer awareness, attitudes and uptake of prevention services (Department of Health, 2007). It is therefore important to ensure that our understanding of knowledge, attitudes and acceptability of the HPV vaccination spans all ethnic groups in the UK and is not just limited to the white population. The aim of this paper is to review the findings of UK-based research that has considered these psychosocial issues in ethnic minority groups.

## Materials and methods

Articles from January 2000 to March 2010 in Medline, CINAHL and PsycINFO were searched using the terms HPV; AND vaccine (or immunisation); AND attitude, aware, barrier, belief, knowledge, perception, psychol^*^ or accept^*^. Reviews, editorials, dissertations, letters and books were excluded. Peer-reviewed journal articles were included if they presented original research, examined knowledge, attitudes or acceptability related to HPV vaccination in a lay population (studies of health professionals were excluded), were carried out in the UK and reported findings by ethnicity.

For the purpose of this review, ethnicity is defined as ‘shared origins or social background; shared culture and traditions that are distinctive, maintained between generations, and lead to a sense of identity and group; and a common language or religious tradition’ (pp 327, Senior and Bhopal, 1994). There are many difficulties with using ethnicity as a variable in epidemiological research and a major one of these is which measurement to use. In the UK Census in 2001, ethnicity was assessed by asking participants which ethnic group they perceived themselves to be in. This ‘self-report’ measure of ethnicity is considered to be an acceptable form of assessment. It allows participants to select different categories at two or more time points, which means it could have limited reliability, however, it is commonly used in research and for the purpose of this review we will consider papers that have measured ethnicity in this way.

There are also many other important ethnicity-related variables including current geographical location, language spoken at home, country of birth, number of years living in the UK and religion. In addition, non-white ethnic groups in the UK tend to be from lower social grades, have lower incomes and are more likely to live in deprived areas. Some minority groups also have lower educational qualifications, although this is not the case across all groups (Nazroo, 2006). Good research exploring ethnicity would therefore take into account a number of the related variables mentioned above. As many of the papers exploring HPV vaccine acceptability have not focused primarily on ethnicity we did not expect this to be the case, hence these variables were not set as part of the inclusion criteria for this review, however, where they have been considered the related findings will be discussed.

## Results

Worldwide 266 articles reported original research (qualitative or quantitative) that examined awareness, knowledge or attitudes related to HPV vaccines, but only 26 of these studies were carried out in the UK. Searching the references of these papers revealed another eight papers. Of the 34 UK-based papers identified 17 were in the ‘lay’ population (parents, adolescents or the general population as opposed to health professionals) and reported findings by ethnicity. Two of these papers were qualitative (Vallely *et al*, 2008; Marlow *et al*, 2009d) and 15 were quantitative (quantitative papers are summarised in Table 1).

### Knowledge about HPV

Between 2001 and 2007, five studies looked at awareness of HPV in the UK (Pitts and Clarke, 2002; Philips *et al*, 2003; Waller *et al*, 2003, 2004; Marlow *et al*, 2007a). Findings pointed to very low levels of awareness with only around 30% of women saying that they had heard of the virus. There was a consistent socioeconomic gradient in HPV awareness, but the small number of non-white participants precluded conclusions about ethnic variation.

In 2007, a study of HPV knowledge recruited participants on the streets of Birmingham, an area with a high proportion of non-white residents (Walsh *et al*, 2008). Purposive sampling was used to ensure a good proportion of ethnic minority participants were included (25% of the sample). Participants were asked six questions relating to their awareness of HPV (e.g. have you heard about HPV, did you know that the government is considering offering HPV vaccination to school girls aged 10–12) and were then allocated a score ranging from 0 (low knowledge) to 6 (high knowledge). Overall, knowledge was extremely poor, with 81% scoring the minimum (i.e. 0). This varied by ethnicity, with 92% of non-white participants scoring ‘0’ compared with 77% of white participants, and this difference was significant even when controlling for gender, age and social class. Those who could not speak English fluently were unable to participate in the survey and this may have resulted in a more acculturated sample, hence true levels of knowledge in the non-white population may have been even lower. In a second Birmingham-based study, published as a short letter to the editor, adolescents aged 12–13 years and their parents were recruited through schools (Das *et al*, 2010). Mean scores on a knowledge scale were 4.54 for non-white and 4.94 for white participants (out of a possible 8). This difference was significant in adjusted analyses, although it is not clear what variables were adjusted for. This study was conducted after introduction of the vaccine, in the target population and the questions included aspects of HPV knowledge that it is important for this population to know e.g. ‘does the vaccine protect against other sexually transmitted infections?’ and ‘is cervical screening necessary after vaccination is given?’ However, because of the publication format there is little detail provided about the methodology, making it difficult to judge the quality of the study.

In another study, quota sampling was used to recruit women from the six main ethnic minority groups in the UK and a white comparison group (Marlow *et al*, 2009c). Interviews were carried out by multilingual interviewers in the language that women spoke at home. Women were asked ‘before this interview, had you heard of HPV?’ Awareness of HPV was lower in all ethnic minority groups: White (39%), Chinese (18%), Caribbean (14%), Pakistani (12%), Indian (9%), African (8%) and Bangladeshi (6%). Religion, migration to the UK and language spoken at home were also considered in this study and each of these variables was a significant predictor of HPV awareness in univariate analyses, but in a multivariate analysis, only ethnicity and religion remained significant. However, the study used quota sampling, hence caution must be exercised when generalising the findings to the wider ethnic minority population.

### Acceptability of HPV vaccination

Seven studies have quantitatively assessed parental acceptability of HPV vaccination in the UK and all suggest that around 74–88% of parents say they would accept HPV vaccination for their daughter (Brabin *et al*, 2006; Marlow *et al*, 2007b, 2008, 2009c; de Visser and McDonnell, 2008; Walsh *et al*, 2008; Morison *et al*, 2010). Findings in relation to ethnic minorities are limited and have been summarised in Table 2.

In 2006/2007 two school-based surveys assessed whether parents thought they would accept HPV vaccination for their daughter (Brabin *et al*, 2006; Marlow *et al*, 2007b). Although there were slightly lower levels of HPV vaccine acceptability for ethnic minorities in both of these studies, the differences were not significant, perhaps because of the small numbers of non-white participants. However, in one of these studies the most acceptable age for HPV vaccination was also considered as an outcome and being from a non-white ethnic group was associated with a higher acceptable age. In multivariate analyses controlling for a range of demographic and attitudinal factors including religion, ethnicity was no longer a significant predictor of acceptable age of vaccination (Marlow *et al*, 2007b). The SES was not associated with acceptability in either of these studies.

In a subset of mothers within a nationally representative survey of British women, there was a 15% difference in the proportion of those who would accept HPV vaccination, between white (76%) and non-white participants (61%), but this finding was not significant, probably due to lack of power (Marlow *et al*, 2008). A study by the same authors that collected data using quota sampling to ensure adequate representation of each UK ethnic minority group, found that although 63% of white mothers said they would accept HPV vaccination, this was much lower among all ethnic minority mothers (11–51%), with the lowest levels in South Asian mothers (11–24%) (Marlow *et al*, 2009c). With more adequate sample sizes these differences were significant but as noted earlier, the quota sampling method makes generalisability to the wider population problematic. Acceptability also varied on the basis of migration status, language spoken at home and religion. In multivariate analyses, which included these variables and a measure of SES, only ethnicity and religion remained significant. Finally, a Birmingham-based street survey asked the more general question: ‘do you think that the introduction of HPV vaccination is a good idea?’ A significant ethnicity effect was found, with white participants showing more positive attitudes about HPV vaccination than non-white participants, (Walsh *et al*, 2008) but the same caveats associated with purposive sampling noted earlier apply.

Two studies have looked at HPV vaccine acceptability in young women aged 16–18 years (the age who were offered the vaccine as part of the catch-up programme) recruited though further education colleges in South East England – both found significant ethnic differences. In the first study, Asian students were less likely to say they would accept HPV vaccination than white students (83 versus 93%), although in multivariate analysis adjusting for religion and language this effect disappeared (Marlow *et al*, 2009b). In a second study using mean intention as the outcome (based on the sum of two intention items), white students had higher intentions to receive the HPV vaccine than those from Asian, Black or other ethnic backgrounds (6.5 *vs* 5.9, 5.7 and 5.6 respectively). Religion was also associated with intention to receive the vaccine, but multivariate analyses were not carried out (Forster *et al*, 2010b). In another study, this time of 14–15-year olds, there were no ethnic differences in intention to accept HPV vaccination (Forster *et al*, 2010a).

All the above studies used acceptability (intention) as their outcome and there is little data on actual HPV vaccination uptake. However, several reports have been published from a feasibility study carried out in two Primary Care Trusts in Manchester in 2007–2008 (Brabin *et al*, 2008, 2009; Stretch *et al*, 2008; Roberts *et al*, 2011). This is the only study in the UK to consider actual uptake of HPV vaccination. Uptake of the first two doses of the vaccine was 69%, but this figure was lower in schools that had a high proportion of ethnic minority children or of children eligible for free school meals (Brabin *et al*, 2008). Overall, 68% of girls received all three doses of the vaccine, but non-white girls were less likely to be fully vaccinated than white girls (odds ratio=0.67, 95% confidence interval: 0.49–0.92) and non-response was more common in the non-white group (odds ratio=1.80, confidence interval: 1.27–2.55). These findings remained when controlling for socioeconomic deprivation. However, a large proportion of the ethnicity data was missing from the sample and had to be imputed (Roberts *et al*, 2011).

### Attitudes to HPV vaccination

Box 1 summarises research findings in relation to HPV vaccine attitudes. Two early qualitative studies explored attitudes to HPV vaccination among white British parents (Noakes *et al*, 2006; Waller *et al*, 2006). The findings suggested that some general concerns about vaccinations, including safety worries and trust in those recommending vaccinations, might be important predictors of HPV vaccine acceptability. In addition, some more specific concerns about HPV vaccination included the sexually transmitted nature of the virus, which some parents felt may give girls *carte blanche* for risky sexual behaviour. A third study used similar qualitative methods, but with purposive sampling to recruit mothers from Black/Black British and Asian/Asian British backgrounds (Marlow *et al*, 2009d). Many of the concerns raised appeared to be consistent with the previous groups of white British parents (e.g. concerns about safety), but there were some additional issues. Mothers seemed to have stronger views about the age of HPV vaccination and this was mainly due to religious beliefs about the unacceptability of sex outside of marriage. They felt that the vaccination would not be necessary for their daughters at 12–13 years and some felt it would not be necessary at all. This is consistent with the quantitative study mentioned earlier that showed mothers from non-white backgrounds had a preference for vaccination against HPV at an older age (Marlow *et al*, 2007b). Mothers also felt the ‘taboo’ surrounding sexually transmitted infections would make it difficult to discuss the vaccination with relatives, and this was something, which they felt was usually an important part of the vaccine decision-making process.

In one quantitative survey that included mothers from a range of ethnic minority groups, reasons were given for intended acceptance of HPV vaccination (Marlow *et al*, 2009c). ‘Protection’ offered by the vaccine was cited less often among Indian (16%), Pakistani (11%), Bangladeshi (13%) and Caribbean mothers (14%) compared with white mothers (29%). Needing more information to make a decision was cited more by Bangladeshi mothers than white British mothers (44% compared with 16%). Sex-related reasons (e.g. ‘it encourages promiscuity’) were more likely to be cited by Indian (15%), Pakistani (20%) and Caribbean mothers (16%) compared with white British mothers (2%). This finding is consistent with another study showing that the belief that HPV vaccination would change sexual behaviour was held more among mothers and adolescents from non-white ethnic groups (Marlow *et al*, 2009a).

There is some evidence that fathers in ethnic minority families may have a more important role in deciding about HPV vaccination (Marlow *et al*, 2009c), with a decision shared by both parents most likely in Indian (61%), Pakistani (57%), Bangladeshi (66%), African (63%) and Caribbean groups (56%) and least likely in Chinese (30%) and white groups (36%). In addition, one study showed that Asian parents were less accepting of letting girls request vaccination at a sexual health clinic without parental consent (Brabin *et al*, 2007).

## Discussion

Few studies have considered ethnic differences in knowledge, attitudes and acceptability of HPV vaccination in the UK. The studies that have been carried out are mostly small and use varied outcome measures, which makes it impossible to combine the findings. There does appear to be consistency across studies, with both knowledge of HPV and acceptability of HPV vaccine being lower for non-white ethnic groups, even if the small sample sizes means these findings are not always significant. Unfortunately categorising ethnicity in this way is far from adequate, and leaves an important question unanswered – for which particular ethnic groups are there inequalities that need to be addressed? Ethnicity researchers have argued for the importance of using meaningful ethnic categories in epidemiological research (e.g. Bhopal, 2006; Nazroo, 2006), but as most of the studies considered in this review did not explicitly aim to explore the role of ethnicity, it is not surprising that they were not designed to include samples large enough to define ethnic categories more appropriately.

Having small numbers of ethnic minority participants also makes it difficult to disentangle ethnicity from other related variables such as language spoken at home, migration status, religion and SES. Understanding whether any of these variables would explain ethnic differences in HPV knowledge and acceptability would offer more insight. Only a few of the studies exploring acceptability included other ethnicity-related variables as potential confounders. Where language spoken at home and migration status were included they did not remain significant in multivariate analyses. However, religion either remained alongside ethnicity or caused the ethnicity effect to disappear from the model, suggesting it has an important role (Marlow *et al*, 2007a,2007b, 2009b,2009c). Interestingly SES did not appear to explain ethnic differences in HPV vaccine acceptability (Marlow *et al*, 2009c) or actual uptake (Roberts *et al*, 2011).

The majority of the work presented here uses acceptability rather than uptake as the outcome. The most recent uptake figures for England showed that overall 76% of eligible girls received all three doses of the vaccine in 2009/2010, with uptake over 80% in many areas (Department of Health, 2010b). This appears to be the case in some areas that are known to have high proportions of ethnic minorities living there (e.g. London borough of Brent: 80%), although not for others (London borough of Newham: 68%). Although there is a box on the vaccine consent form for parents to indicate their ethnicity, these data have not been made available (and may be very incomplete), hence estimates of variation in uptake by ethnic group rely on school or area level data. The hope is that this paper will inspire the continuation of research in the actual vaccination context.

This review also considered studies exploring knowledge of and attitudes towards HPV and the vaccine. Although knowledge is not closely correlated with acceptability, it was included because of the implications it has for informed consent. Ensuring people are well informed before making a health-related decision is expected to result in better patient outcomes, and respects the need to consider ethical principles and potential litigation (Marteau *et al*, 2001). Only three studies considered knowledge of HPV by ethnicity, and their findings suggested lower awareness of HPV and lower knowledge scores based on a range of relevant items (Walsh *et al*, 2008; Marlow *et al*, 2009c; Das *et al*, 2010). It is well established that attitudes are correlated with vaccine uptake, however, very few studies have considered attitudes towards HPV vaccination among ethnic minorities in the UK.

There is a need for more research that aims to explore HPV knowledge, attitudes and vaccination uptake among ethnic minorities, in the context of the actual vaccination programme. Future studies in this context will need to consider their methodology carefully. In order to make comparisons across a range of ethnic groups, very large samples of each group are needed. Quota sampling helps to overcome this problem, and was used in two of the studies described above (Walsh *et al*, 2008; Marlow *et al*, 2009c), but there are limitations to this method, with participants likely to be self-selected and not necessarily representative of the minority population. Using data from the HPV immunisation programme could overcome this bias and would ensure a large sample. One study in the Netherlands used this method, evaluating determinants of HPV vaccine uptake nationwide. Ethnic minority groups (recorded as parent's country of birth) only represented a small proportion of the sample, but the large sample size (384 869) meant they were able to look at ethnic differences and control for possible confounders (Rondy *et al*, 2010). Unfortunately ethnicity data is often missing from such records (Rondy *et al*, only had ethnicity data for 28% of adolescents). This means only smaller sub-samples can be included in analyses or ethnic data have to be imputed (as in Roberts *et al*, 2011). Another option is to recruit in the same way as a nationally population representative survey (i.e. by using lists such as the Postcode Address File) but to use ‘ethnic boosts,’ oversampling areas that have high proportions of ethnic minorities. This method is used in surveys such as the Health Survey for England, but is a very expensive method of data collection. Alternatively researchers may wish to focus their work on one or two ethnic groups.

## Conclusion

We know relatively little about attitudes to HPV vaccination in ethnic minorities in the UK. Findings seem to suggest lower awareness of HPV and lower acceptability of the vaccination, which could be important if they are reflected in uptake. Only two studies have focused specifically on ethnic minorities in the UK (Marlow *et al*, 2009c, 2009d), and the others have looked at ethnicity as just one of many factors. It is important to include ethnic minorities in cancer prevention research to ensure that culturally specific barriers are identified and potential ethnic inequalities are addressed. More research is needed, particularly in the context of the vaccination programme. Uptake figures so far suggest that the vaccine is generally well received, but details of ethnic variation in these figures are still being awaited.

## Figures and Tables

**Table 1:** Summary of quantitative studies that have assessed ethnic differences in knowledge and acceptability of HPV vaccination in the UK

**Study**	**Study design (participants)**	**Ethnic breakdown of sample**	**Outcomes**	**Main ethnicity-related findings**	**Multivariate analyses**
Brabin *et al* (2006)	School-based survey, adjusted for sampling (parents, *n*=317, RR=22%).	White (66%), Indian (13%), Black African (9%), Black Caribbean (8%), others (4%).	Acceptability for daughter (single item).	Ethnicity and religion were not associated with acceptability.	
Brabin *et al*, (2007)	School-based survey, adjusted for sampling (parents, *n*=317, RR=22%).	White (66%), Indian (13%), Black African (9%), Black Caribbean (8%), others (4%).	Attitude to vaccinating a well-informed child against HPV without parental consent (qualitative responses coded as positive, less positive or other).	White and Black Caribbean parents were more supportive of vaccinating without parental consent than Black African or Indian parents. Religion was not associated with attitude to consent.	
Marlow *et al* (2007a)	A nationally representative sample (*n*=1620, RR=53%).	White (94%), Non-white (6%).	Awareness of HPV (single prompted item).	Ethnicity was not associated with awareness of HPV.	
Marlow *et al* (2007b)	School-based survey (parents, *n*=684, 57%).	White (94%), Non-white (6%).	Acceptability for daughter (single item). Most acceptable age for vaccination (single item).	Ethnicity was not associated with acceptability. Those from ‘other’ religions were less accepting. Ethnicity and religion were associated with selecting an older age for HPV vaccination.	The effect of ethnicity on acceptable age for HPV vaccination disappeared when controlling for other factors. Religion remained significant.
Marlow *et al* (2008)	Sub-set of mothers with a daughter <16 years, within a nationally representative sample (*n*=296, RR=53%).	White (90%), Non-white (10%).	Acceptability of HPV vaccination (single item).	Ethnicity was not associated with acceptability.	
Brabin *et al* (2008)	Vaccine feasibility study in two PCTs in Manchester (*n*=2817).	School-based data on ethnic proportion in each school.	Uptake of the first two doses of the vaccine.	Uptake was lower in schools that had higher proportions of ethnic minorities and those eligible for free school meals.	
Walsh *et al* (2008)	Street survey, purposive sampling (men and women, *n*=420).	White (73%), Asian (13%), Black (9%), Mixed (3%), Other (3%).	Knowledge (6-point knowledge score). Attitudes (single item: do you think that the introduction of HPV vaccination is a good idea?).	Non-white participants were more likely to score 0. Non-white participants showed less positive attitudes to HPV vaccination.	Ethnic differences in knowledge remained when controlling for gender, age and social class. Ethnic differences in attitudes remained when controlling for age (SES was not associated with attitudes, so not controlled for).
Stretch *et al* (2008)	Parents in a vaccine feasibility study who had agreed to be sent questionnaires (*n*=651, RR=60%).	Not reported.	Having consented or refused HPV vaccination for their daughter.	There was no association between ethnicity and consenting/refusing the HPV vaccination, However, non-white parents were less likely to have returned the questionnaire.	
Marlow *et al* (2009a)	A sub-set of mothers from a nationally representative sample who had a daughter under 16 years (*n*=332, RR=54%) and a school-based study of 16–18-year-old girls (*n*=328).	Mothers: White (91%), Black (4%), Asian (4%). Adolescent girls: White (59%), Black (24%), Asian (13%), other (4%).	Anticipated risk compensation (sum of two items relating to more sex or unprotected sex following HPV vaccination), girls also completed questions relating to their own behaviour.	Compared with white participants, Black and Asian mothers and girls from Black backgrounds had higher risk compensation scores. Among girls there was no association between ethnicity and belief about changes in own sexual behaviour.	
Marlow *et al* (2009c)	Face-to face interviews, quota sampling (women only, *n*=950, RR=70%).	Indian (25%), White (21%), Pakistani (17%), Caribbean (14%), African (11%), Bangladeshi (7%), Chinese (6%).	Awareness of HPV (have you heard of HPV?). Acceptability for a daughter in subset of mothers.	Ethnic minority participants were less likely to say they had heard of HPV. Religion, migration status and language were also associated with awareness. Ethnic minority participants were less likely to say they would accept HPV vaccination. Religion, migration status and language were also associated with acceptability.	In multivariate analyses ethnicity and religion remained significant of HPV awareness. For HPV vaccine acceptability, ethnicity and religion were significant in multivariate analyses, which included SEC and the other ethnicity-related variables.
Marlow *et al* (2009b)	School-based survey (16–18-year olds, *n*=328).	White (58%), Asian (13%), Black (25%), other (4%).	Acceptability of HPV vaccination (single item).	Students from Asian backgrounds were less likely to say they would accept HPV vaccination. Religion and language spoken at home were also associated with acceptability.	In multivariate analyses with ethnicity, religion and language spoken at home, only religion was significant.
Das *et al* (2010)	School-based survey (12–13-year olds and their parents, *n*=434).	White (72%), Asian (18%), Mixed (6%), Black (3%), other (1%).	Knowledge (8-point knowledge score).	Mean knowledge scores were lower for non-white participants.	
Forster *et al* (2010b)	School-based (16–18-year olds, *n*=617, 94%).	White (55%), Asian (19%), Black (12%), other (11%).	Acceptability of HPV vaccine (sum of two items).	Ethnicity and religion were associated with acceptability.	
Forster *et al* (2010a)	School-based (14–15-year olds, *n*=173).	White (73%), Asian (11%), other (11%), non-response (6%).	Intention to receive HPV vaccination (single item).	Neither ethnicity nor religion was associated with intention to receive HPV vaccination.	
Roberts *et al* (2011)	Vaccine feasibility study in two PCTs in Manchester (*n*=2817).	White (91%), Asian (5%), Black (1%), mixed (3%), other (1%).	Three groups: uptake of all three doses of the vaccine, active refusal or non-response.	Non-white girls were less likely to be fully vaccinated and more likely to be non-responders.	These effects remained when controlling for deprivation score.

Abbreviations: HPV=human papillomavirus; PCT=Primary Care Trust; RR=response rate, which is reported where possible.

**Table 2:** Studies that have looked at ethnic variation in parental acceptability of HPV vaccination

**Study**	**Ethnic groupings (*n*)**	**Acceptability %**
Brabin *et al* (2006)	White (207)	81
	Black Caribbean (25)	56
	Black African (28)	71
	Indian (39)	67
	Others (13)	85
Marlow *et al* (2007a, 2007b)	White (617)	76
	Non-white (42)	64
Walsh *et al* (2008) a	White (305)	91
	Non-white (115)	81
Marlow *et al* (2008)	White (269)	76
	Non-white (31)	61
Marlow *et al* (2009a, 2009b, 2009c, 2009d)^a^	White British (149)	63
	Pakistani (112)	11
	Bangladeshi (45)	18
	Indian (130)	25
	Chinese (30)	40
	Caribbean (74)	49
	African (61)	51

Abbreviation: HPV=human papillomavirus.

a Mean significant differences were found.

## Summary of attitudes to HPV vaccination

*Attitudes to HPV vaccination that span across all ethnicities:* Concerns about safety and side effects Novelty of the vaccination Discussing with others

*Attitudes with some evidence of particular relevance to some ethnic minorities:* Concerns about changes in sexual behaviour Low perceived risk

*Attitudes that are culturally specific:* Concern about genetic differences Taboo topic Religious beliefs
